# Supplementary material for: Development and validation of an interpretable model integrating multimodal information for improving ovarian cancer diagnosis
Source: Nat Commun. 2024 Mar 27;15:2681. doi: 10.1038/s41467-024-46700-2 (PMC10973484; doi:10.1038/s41467-024-46700-2)
Supplement: Supplementary file 1 — Supplementary Information [file 41467_2024_46700_MOESM1_ESM.pdf]

# Development and Validation of an Interpretable Model Integrating Multimodal Information for Improving Ovarian Cancer Diagnosis

**Xiang et al**

**Supplementary Table 1. The performances of different models in patient level.**

| Internal test set | DenseNet121 | DenseNet169 | DenseNet201 | ResNet34 | EfficientNet_b5 | EfficientNet_b6 | Ensemble |
|---|---|---|---|---|---|---|---|
| AUC | 0.923(0.904, 0.944) | 0.915(0.895, 0.931) | 0.911(0.891, 0.929) | 0.898 (0.874, 0.924) | 0.915(0.898, 0.932) | 0.911(0.888, 0.930) | 0.970(0.934, 0.993) |
| Sensitivity (%) | 79.4(75.4, 83.2) | 84.5(80.9, 87.7) | 83.5(79.9, 87.0) | 80.7(76.9, 85.0) | 89.2(86.0, 92.2) | 83.2(79.7, 86.7) | 86.7(78.7, 94.7) |
| Specificity (%) | 91.6(88.2, 94.6) | 85.9(81.8, 89.9) | 86.9(83.2, 90.6) | 85.2(80.8, 89.2) | 77.2(72.4, 8.15) | 85.6(81.1, 89.2) | 98.1(94.3, 100.0) |
| Accuracy (%) | 84.6(81.9, 86.9) | 85.1(82.5, 87.8) | 84.9(82.3, 87.6) | 82.6(79.9, 85.3) | 84.1(81.2, 86.5) | 84.2(81.3, 87.1) | 91.5(86.7, 96.1) |
| PPV (%) | 92.7(90.1, 95.3) | 88.9(86.2, 91.8) | 89.5(86.9, 92.3) | 88.0(85.0, 91.0) | 84.0(81.3, 86.7) | 88.5(85.5, 91.3) | 98.5(95.5, 100.0) |
| NPV (%) | 76.9(73.4, 80.1) | 80.5(77.0, 84.1) | 79.7(76.1, 83.6) | 76.7(73.1, 80.7) | 84.2(80.1, 88.0) | 79.2(75.5, 83.0) | 84.1(76.8, 92.7) |
| External test set | | | | | | | |
| AUC | 0.806(0.787, 0.825) | 0.817(0.792, 0.838) | 0.818(0.795, 0.840) | 0.821(0.803, 0.845) | 0.848(0.825, 0.865) | 0.851(0.831, 0.869) | 0.893(0.850, 0.931) |
| Sensitivity (%) | 76.0(72.4, 79.6) | 73.6(70.2, 77.1) | 67.1(62.7, 71.3) | 79.8(76.7, 83.1) | 79.3(75.6, 82.9) | 72.2(68.7, 75.8) | 77.8(66.7, 86.4) |
| Specificity (%) | 71.5(69.4, 73.7) | 75.8(73.7, 77.9) | 82.5(80.5, 84.6) | 71.4(69.4, 73.5) | 74.4(72.1, 76.4) | 83.8(82.0, 85.4) | 89.5(86.3, 92.8) |
| Accuracy (%) | 72.6(71.0, 74.6) | 75.3(73.5, 77.1) | 78.7(76.9, 80.5) | 73.5(71.8, 75.3) | 75.6(73.8, 77.2) | 80.9(79.4, 82.5) | 87.1(84.0, 90.2) |
| PPV (%) | 47.1(45.1, 49.5) | 50.4(48.0, 53.0) | 56.2(53.1, 59.5) | 48.2(46.2, 50.3) | 50.8(48.4, 52.9) | 59.7(57.0, 62.6) | 66.3(59.4, 74.4) |
| NPV (%) | 89.9(88.6, 91.3) | 89.6(88.4, 90.9) | 88.3(87.0, 89.6) | 91.4(90.2, 92.7) | 91.5(90.1, 92.9) | 90.0(88.9, 91.3) | 93.8(91.2, 96.0) |

Note: Data in parentheses are 95% confidence intervals; AUC, area under the receiver operating characteristic curve; PPV, positive predictive value; NPV, negative predictive value. Cut-off thresholds of each model are corresponding to the maximum of Youden index.
