# Supplementary material for: Development and validation of an interpretable model integrating multimodal information for improving ovarian cancer diagnosis
Source: Nat Commun. 2024 Mar 27;15:2681. doi: 10.1038/s41467-024-46700-2 (PMC10973484; doi:10.1038/s41467-024-46700-2)
Supplement: Supplementary file 3 — Reporting Summary [file 41467_2024_46700_MOESM3_ESM.pdf]

nature portfolio

Corresponding author(s): Xi Lin & Hao Chen

Last updated by author(s): Feb 8, 2024

Reporting Summary

Nature Portfolio wishes to improve the reproducibility of the work that we publish. This form provides structure for consistency and transparency in reporting. For further information on Nature Portfolio policies, see our Editorial Policies and the Editorial Policy Checklist.

Statistics

For all statistical analyses, confirm that the following items are present in the figure legend, table legend, main text, or Methods section.

n/a	Confirmed	
☐	☒	The exact sample size (n) for each experimental group/condition, given as a discrete number and unit of measurement
☐	☒	A statement on whether measurements were taken from distinct samples or whether the same sample was measured repeatedly
☐	☒	The statistical test(s) used AND whether they are one- or two-sided<br>*Only common tests should be described solely by name; describe more complex techniques in the Methods section.*
☒	☐	A description of all covariates tested
☒	☐	A description of any assumptions or corrections, such as tests of normality and adjustment for multiple comparisons
☐	☒	A full description of the statistical parameters including central tendency (e.g. means) or other basic estimates (e.g. regression coefficient) AND variation (e.g. standard deviation) or associated estimates of uncertainty (e.g. confidence intervals)
☐	☒	For null hypothesis testing, the test statistic (e.g. F, t, r) with confidence intervals, effect sizes, degrees of freedom and P value noted<br>*Give P values as exact values whenever suitable.*
☒	☐	For Bayesian analysis, information on the choice of priors and Markov chain Monte Carlo settings
☒	☐	For hierarchical and complex designs, identification of the appropriate level for tests and full reporting of outcomes
☒	☐	Estimates of effect sizes (e.g. Cohen's d, Pearson's r), indicating how they were calculated

Our web collection on statistics for biologists contains articles on many of the points above.

Software and code

Policy information about availability of computer code

Data collection	GE Logiq S8 (GE Healthcare, Milwaukee, WI, USA) or Aplio 300, 400, or 500 (Toshiba Medical System, Tokyo, Japan) systems.
Data analysis	Data analysis was performed with R (version 3.6.3).

For manuscripts utilizing custom algorithms or software that are central to the research but not yet described in published literature, software must be made available to editors and reviewers. We strongly encourage code deposition in a community repository (e.g. GitHub). See the Nature Portfolio guidelines for submitting code & software for further information.

Data

Policy information about availability of data

All manuscripts must include a data availability statement. This statement should provide the following information, where applicable:

- Accession codes, unique identifiers, or web links for publicly available datasets
- A description of any restrictions on data availability
- For clinical datasets or third party data, please ensure that the statement adheres to our policy

The private data analysed in this study are not publicly available due to hospital regulations and patient privacy considerations. All data generated or analysed during the study are included in the published paper. The codes are available at the web repository of "https://github.com/Xiao-OMG/OvcaFinder", which can be used for only non-commercial research purpose.

Research involving human participants, their data, or biological material

Policy information about studies with human participants or human data. See also policy information about sex, gender (identity/presentation), and sexual orientation and race, ethnicity and racism.

Reporting on sex and gender	All participants enrolled are female.
Reporting on race, ethnicity, or other socially relevant groupings	Participants were exclusively collected in China. The model's performance in ovarian cancer diagnosis in other ethnicities merits further investigation.
Population characteristics	Statistics about cohorts was shown in Table 1.
Recruitment	Patients were eligible if they presented with at least one pathology-proven adnexal lesion visible on TVUS examination. To ensure a complete evaluation, transabdominal examinations were included if the lesions were too large to be fully evaluated by TVUS. When multiple lesions were detected, the lesion with the most complex morphology was chosen for analysis. If lesions had similar features, the largest one was included.
Ethics oversight	This study was approved by the Institutional Review Board of Sun Yat-sen University Cancer Center (B2022-112-01).

Note that full information on the approval of the study protocol must also be provided in the manuscript.

Field-specific reporting

Please select the one below that is the best fit for your research. If you are not sure, read the appropriate sections before making your selection.

☒ Life sciences ☐ Behavioural & social sciences ☐ Ecological, evolutionary & environmental sciences

For a reference copy of the document with all sections, see nature.com/documents/nr-reporting-summary-flat.pdf

Life sciences study design

All studies must disclose on these points even when the disclosure is negative.

Sample size	No sample size calculation was performed. We included patients as many as possible after excluding those who did not meet the inclusion criteria.<br>SYSUCC: 724 patients<br>CQUCC: 387 patients
Data exclusions	The exclusion criteria were: (1) physiological changes, such as a follicle or corpus luteum with a diameter less than 3cm in premenopausal women; (2) a prior diagnosis of ovarian cancer; (3) loss of clinicopathological information; or (4) a time interval between ultrasound examination and biopsy or surgery exceeding 120 days.
Replication	The model was developed using the training set in SYSUCC. Then it was internally and externally validated with the internal test set from SYSUCC and the external CQUCC set, respectively. There was no overlaps among the aforementioned datasets.
Randomization	The images from SYSUCC were randomly divided into training, validation, and internal test sets at a ratio of 7:1:2.
Blinding	In the reader study, readers independently assessed all anonymised and randomised lesions, blinded to all clinicopathological information.

Reporting for specific materials, systems and methods

We require information from authors about some types of materials, experimental systems and methods used in many studies. Here, indicate whether each material, system or method listed is relevant to your study. If you are not sure if a list item applies to your research, read the appropriate section before selecting a response.

Materials & experimental systems

n/a	Involved in the study	
☒	☐	Antibodies
☒	☐	Eukaryotic cell lines
☒	☐	Palaeontology and archaeology
☒	☐	Animals and other organisms
☒	☐	Clinical data
☒	☐	Dual use research of concern
☒	☐	Plants

Methods

n/a	Involved in the study	
☒	☐	ChIP-seq
☒	☐	Flow cytometry
☒	☐	MRI-based neuroimaging
